# An anti-clogging method for improving the performance and lifespan of blood plasma separation devices in real-time and continuous microfluidic systems

**DOI:** 10.1038/s41598-018-35235-4

**Published:** 2018-11-19

**Authors:** Dong-Hyun Kang, Kyongtae Kim, Yong-Jun Kim

**Affiliations:** School of Mechanical Engineering, Yonsei University, 50, Yonsei-ro, Seodaemun-gu, Seoul 03722 Republic of Korea

## Abstract

On-chip blood plasma separators using microfluidic channels are typically developed as disposable devices for short-term use only because blood cells tend to clog the microchannels, limiting their application in real-time and continuous systems. In this study, we propose an anti-clogging method. We applied dielectrophoresis to prevent microchannel clogging in a plasma separator that can be used over long periods for real-time and continuous monitoring. Prior to applying the anti-clogging method, the blood plasma separator stopped working after 4 h. In contrast, by manipulating the separator with the new anti-clogging method at a voltage of 20 V, it continued working in a long-term experiment for 12 h without performance deterioration or an increase in cell loss. Two critical performance parameters of the manipulated separator, the purity efficiency and the plasma yield, were 97.23 ± 5.43% and 38.95 ± 9.34%, respectively, at 20 V after 15 min. Interestingly, the two performance parameters did not decrease during the long-term experiment. Hence, the blood plasma separator with the anti-clogging method is an interesting device for use in real-time and continuous blood plasma separation systems because of its consistent performance and improved lifespan.

## Introduction

Human blood performs many critical function for the body by supporting processes like nourishing tissues, regulating organ activities, and defending against harmful agents. Plasma, which is the liquid component of blood that suspends blood cells and many substances, constitutes more than 50% of the blood volume. Plasma serves in a variety of functions from maintaining the blood pressure and volume to transporting critical proteins involved in blood clotting and immunity^1,2^. It also serves as the medium for exchange of vital minerals such as sodium and potassium and helps maintain a proper pH balance in the body^2^. Plasma can be rich with indicators of various diseases, which is why separating plasma from blood is of clinical importance^1–3^.

Conventional methods for plasma separation use centrifugation, which specifically supports processing of large volumes of blood. Although the conventional methods are very efficient and most commonly used in research and clinical laboratories, they have many limitations including the need for highly skilled personnel to operate the high-cost equipment and analyse the results. To circumvent this limitation, the lab-on-a-chip (LOC) approach by miniaturization and integration of the blood plasma separation procedure has gained an increasing interest in the past few years^4–15^. The approach offers many advantages, such as the use of very small quantities of samples and reagents, a high resolution and sensitivity in separation and detection methods, low cost, short analysis times, and a small footprint for the analytical devices. Numerous microfluidic-based on-chip devices and techniques have been proposed for blood plasma separation, such as capillary force^4^, geometrical obstacles^5,6^, sedimentation^7,8^, acoustic forces^9^, inertial forces^10,11^, micro-filtration^12,13^, magnetophoresis^14^, and electroosmotic flow^15^. Some of the on-chip blood plasma separation methods are efficient. However, clogging is an issue for applications with long separation time in continuous systems^16–18^. In miniaturized plasma extraction systems, blood cells and plasma are typically separated by microfluidic channels. Within a microfluidic channel, blood cells tend to move along the axis of the microchannel, thereby increasing the cell concentration along the centre of the microchannel. When a microfluidic channel splits into more than two branches for blood plasma separation, the fluid velocity increases because of the shrinking of the cross-sectional areas in the microchannel. Therefore, blood cells located near the centre of the microchannel are accelerated by the fluid velocity and approach the microchannel wall because of their inertia^19,20^. This process is accelerated because of the high cell content in blood^19^.

The phenomenon of blood cell adherence to the microchannel wall is termed a cell loss in this paper. It causes performance and reliability deterioration and unexpected structural changes or system pressure drops. Hence, microchannels are very prone to clogging during blood processing because of the extremely high number of cells and the strong adhesive force caused by a large surface-to-volume ratio. Once blood cells adhere to the microchannel wall, it is difficult to detach them. Some research groups already described ‘clogging-free’ blood plasma separation methods^21–23^. However, these reports did not mention how long and how often the device can be used, and whether it can be only operated using specifically designed microchannel. Moreover, some groups used blood with very low haematocrit^21,23^. For anti-clogging of the microchannel, most research groups have used surfactant or surface treatment^24^. However, using a surfactant, which is a chemical method, pollutes the medium. The surface treatment method is not suitable for a device that is operated for a long time due to the problem of durability.

Hence, the objective of this study was to develop a microfluidic channel device with a long lifespan for continuous, real-time blood plasma separation using an anti-clogging method. In this study, we applied dielectrophoresis (DEP) to a hydrodynamic blood plasma separation device as anti-clogging technique that does not damage microfluidic devices. In addition, this technique can be applied to most microchannel devices without any restriction regarding the channel design. To verify the effect of the anti-clogging technique and the prevention of cell loss, interdigitated (IDT) electrode pairs were integrated into the device at the bottom of the branch channel for plasma extraction. Moreover, we tested if the technique improves the purity efficiency and the plasma yield.

## Background

### Cell adhesion and clogging in microchannel

**Figure 1:**
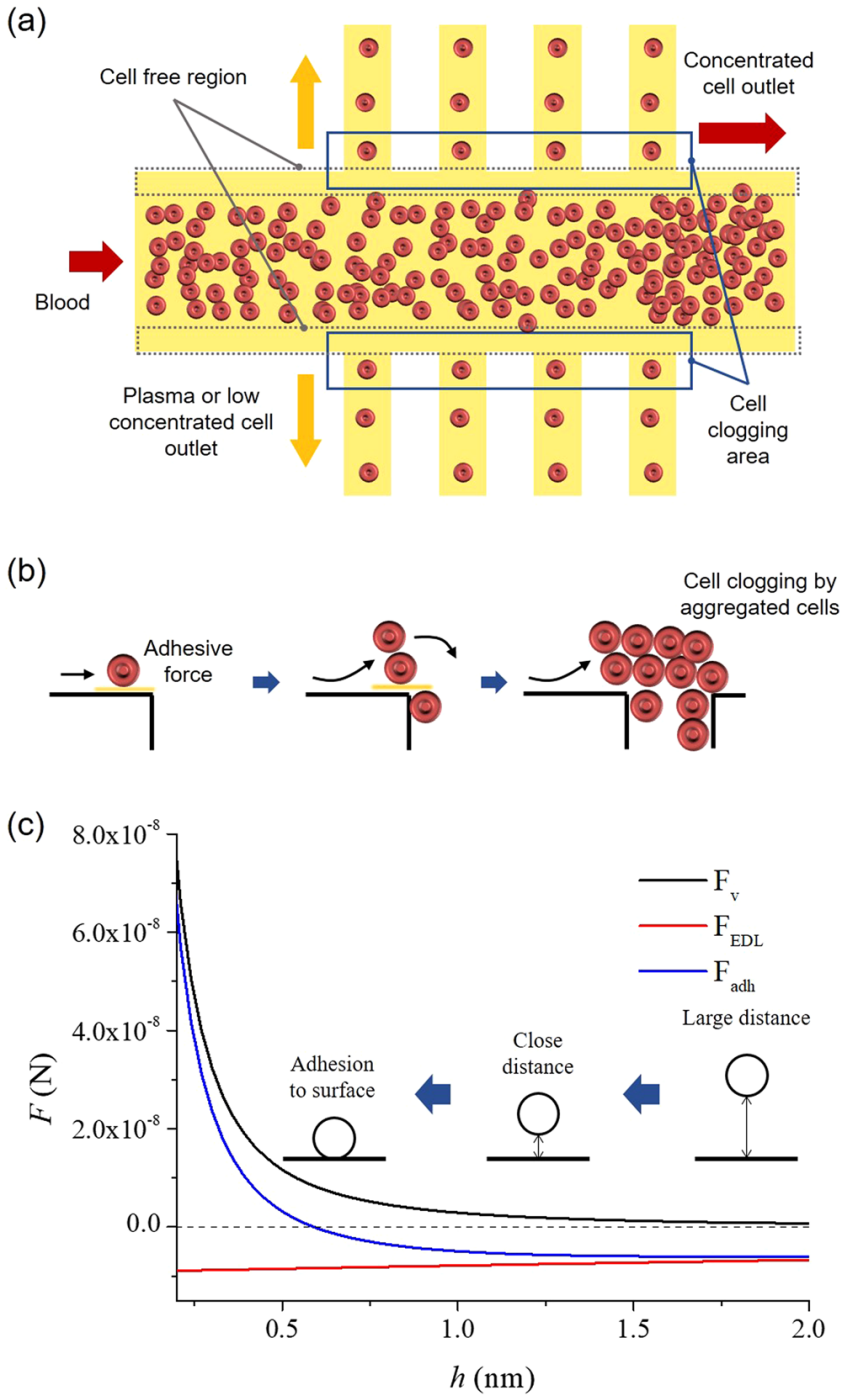
The mechanism of cell aggregation and clogging in a microchannel device. (**a**) Schematic presentation of a microchannel arrangement for blood plasma separation. If blood moves through the microchannel, RBCs, in particular, tend to migrate towards the axis of the microchannel, thereby increasing the cell concentration along the centre. In contrast, cell-free regions appear near the microchannel wall. Therefore, most of the on-chip blood plasma separation devices for continuous monitoring separate the blood plasma by extracting the cell-free regions. However, some cells also move through the extracting microchannel as undesirable material. (**b**) Schematic representation of the sequence of events during aggregation and clogging in a microchannel from the initial cell deposition to the clog formation. (**c**) The adhesive force (sum of the van der Waals force and the electrostatic double layer force) in relation to the distance between a cell and the surface. If a cell gets closer to the surface, the adhesive force sharply increases (*h* < 0.5 nm).

Typically, on-chip blood plasma separation devices have a microfluidic network consisting of two or more split and branched microchannels that promote the blood plasma separation because of hydrodynamic forces^5–8,10,11^. As shown in Fig. 1a, the branched microchannels have a very narrow width to facilitate plasma extraction. The fluid flow is accelerated where the microchannel splits into more than two narrow branch channels because of the difference in channel dimensions. At the same time, blood cells, for example red blood cells (RBCs), white blood cells (WBCs), and platelets (PLTs), approach the microchannel wall because of the change in fluid flow. To identify locations of cells in splitted microchannel, a simulation was performed based on the simple compressible Navier-Stokes equation in the Eulerian reference frame (shown in Supplementary Fig. 1). At the entrance of the branch channels, blood cells are attached to the microchannel wall because of the adhesive force near the microchannel wall (shown in Fig. 1b). When the cells start to attach to the branch channel wall, they reduce the channel diameter and increase the diameter difference between the main and the branch channel. Therefore, the cell attachment to the channel wall surface is dramatically accelerated and not only by cell-to-surface attachment but also by cell-to-cell attachment (aggregation), causing microchannel clogging. Consequently, the adhesion of a single cell to the microchannel wall initiates aggregation and clogging.

The adhesive force between a single cell and a solid surface is critical for the mechanism of microchannel clogging. Typically, when a blood cell is in close proximity to a planar surface, it is pulled to an adhesive contact with the planar surface by physical and chemical surface forces at the interface^25,26^. The interactions are a combination of attraction by van der Waals forces and repulsion by electrostatic double layer forces, which are acting on relatively long-range^25–27^. After the initial contact, the cells attach to the surface more strongly through specific cell membrane factors that selectively bind the cell to the surface^19,20,27^.

In this study, we aimed to prevent the initial cell attachment to the internal microchannel surface. We hypothesized that the long-range adhesive forces which are critical for disturbing initial cell attachment.

The adhesive force *F*_adh_ on the single cell is composed of the van der Waals force *F*_v_ and the electrostatic double layer force *F*_EDL_^19,20^.

1 $${F}_{{\rm{adh}}}={F}_{{\rm{v}}}+{F}_{{\rm{EDL}}}$$

The surface of the microchannel wall can be considered flat and the two forces are normal on the surface. The strength of these forces depends on the distance *h* between the cell and the surface and on the surface properties. The two forces are described as follows.

2 $${F}_{{\rm{v}}}=\frac{Ad}{12{h}^{2}}$$

3 $${F}_{EDL}=32\pi {\varepsilon }_{{\rm{m}}}\kappa d{\gamma }_{{\rm{p}}}{\gamma }_{{\rm{w}}}\exp (\,-\,\kappa h){(\frac{{k}_{{\rm{B}}}T}{e})}^{2}$$

*A* is the Hammaker constant (5 zJ)^20^, *d* is the particle diameter, *ε*_m_ is the permittivity of the medium (78 *ε*_0_)^28^, *κ* is the Debye parameter (0.159 nm^−1^)^29^, *k*_B_ is the Boltzmann constant (1.38 × 10^−23^ J/K), *T* is the absolute temperature (296.15 K), and *e* is the fundamental electronic charge (1.602 × 10^−19^ C). The terms *γ*_p_ and *γ*_w_ are defined as *γ*_p/w_ = tanh[(*zeζ*_p/w_)/(4*k*_B_*T*), respectively^19^, where *ζ*_p/w_ is the surface zeta potential (RBCs: −10.8 mV and PDMS: −13.825 mV) and *z* the valency of the symmetric electrolyte^29,30^.

The adhesive force on a cell increases sharply close to the microchannel wall surface (*h* < 0.5 nm), as shown in Fig. 1c. The attractive van der Waals forces cause the cell to spontaneously attach to the surface. At the entrance of branch channels, initial cell attachment occurs because of the strong adhesion forces at the surface whereas the following cell aggregations are driven by cell-to-cell adhesion, which accelerate over time, as shown in Fig. 1b. Therefore, the microchannels are clogged by aggregated and attached cells, which will be prevented by blocking the initial cell attachment.

### Anti-clogging method

**Figure 2:**
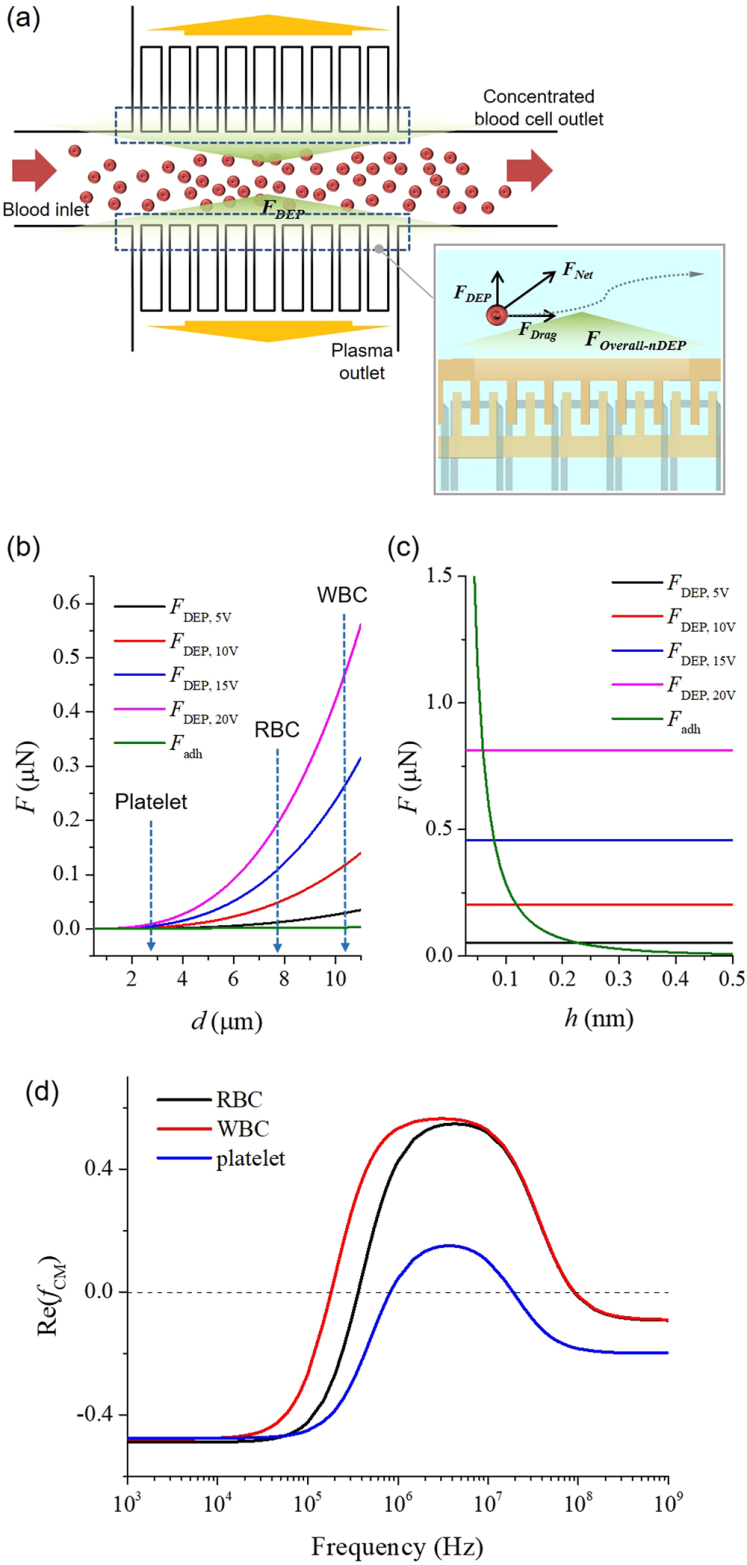
Optimisation of anti-clogging method. (**a**) Schematic view of the proposed anti-clogging method for long term use of blood plasma separation. At the entrances of the branch microchannels for blood plasma separation, nDEP was generated, which blocked blood cells from approaching the channel wall. (**b**) The adhesive and DEP force were plotted against the particle diameter range. The DEP force was greater than the adhesive force (green graph) on blood cells. (**c**) The adhesive and DEP force were plotted against the distance between the cells (RBCs: 7 µm) and the surface of the microchannel wall (*h*). At *h* < 0.5 nm, the adhesive force (green graph) increased sharply. However, at *h* > 0.5 nm, the DEP force was higher than the adhesive force. (**d**) The real part of the Clausius-Mossotti factor of RBC, WBC, and PLT as various frequency. The real part of the Clausius-Mossotti factor was about −0.5 for an applied electrical frequency of <10 kHz for blood cells.

To ensure the anti-clogging effect, DEP was used as a repulsive force for disturbing the initial cell interaction with the microchannel surface as shown in Fig. 2a. DEP is a molecular migration phenomenon that occurs in the presence of polarizable particles within an inhomogeneous electric field^28^. Inhomogeneous electric fields, which may be generated by electrodes in a microfluidic system, induce electric dipole moments in particles like blood cells. The repulsive force, the DEP force, exerted on particles *F*_DEP_ can be expressed as follows^28^.

4 $${F}_{{\rm{DEP}}}=2\pi {r}^{3}{\varepsilon }_{{\rm{m}}}{\rm{Re}}({f}_{{\rm{CM}}})\nabla {({E}_{{\rm{rms}}})}^{2}$$

The *r* is the particle radius, *ε*_m_ is the permittivity of the medium, Re(*f*_CM_) is the real part of the Clausius-Mossotti factor, and *E*_rms_ is the root mean square value of the electric field. The Clausius-Mossotti factor *f*_CM_, determined by the complex permittivity of the particle ε*_p_ and the medium ε*_m_, is a function of the frequency of the electric field.

5 $${f}_{{\rm{CM}}}=\frac{{\varepsilon }_{{\rm{p}}}^{\ast }-{\varepsilon }_{{\rm{m}}}^{\ast }}{{\varepsilon }_{{\rm{p}}}^{\ast }+2{\varepsilon }_{{\rm{m}}}^{\ast }}$$

The complex permittivity can be written as follows.

6 $${\varepsilon }^{\ast }=\varepsilon -j\frac{\sigma }{\omega }$$

The *ω* is the frequency of the electric field, and *σ* is the electric conductivity.

The real part of the Clausius-Mossotti factor, Re(*f*_CM_), ranges from −0.5 to 1.0 and determines the direction of the DEP force exerted on particles^28,31,32^. When Re(*f*_CM_) is positive, it is called positive DEP (pDEP); and particles move toward high electric field density. Conversely, when Re(*f*_CM_) is negative, it is called negative DEP (nDEP); and particles move toward low electric field density^28^. In this study, nDEP was used as repulsive force to prevent cell loss and clogging in the microchannels. The real part of the Clausius-Mossotti factor is related to the permittivity and conductivity of the particles along with those of the medium and the frequency of the electric field. However, because biological cells, like RBCs and WBCs, are not homogeneous spheres, the high contrast of conductivity between cytoplasm and the isolating membrane makes it necessary to consider a core-shell model. To obtain the effective complex permittivity of cells, a mixing equation was developed^28^.

7 $${\varepsilon }_{{\rm{effective}}}^{\ast }={\varepsilon }_{{\rm{outer}}}^{\ast }\cdot \frac{{(\frac{{r}_{{\rm{outer}}}}{{r}_{{\rm{inner}}}})}^{3}+2\frac{{\varepsilon }_{{\rm{inner}}}^{\ast }-{\varepsilon }_{{\rm{outer}}}^{\ast }}{{\varepsilon }_{{\rm{inner}}}^{\ast }+2{\varepsilon }_{{\rm{outer}}}^{\ast }}}{{(\frac{{r}_{{\rm{outer}}}}{{r}_{{\rm{inner}}}})}^{3}-\frac{{\varepsilon }_{{\rm{inner}}}^{\ast }-{\varepsilon }_{{\rm{outer}}}^{\ast }}{{\varepsilon }_{{\rm{inner}}}^{\ast }+2{\varepsilon }_{{\rm{outer}}}^{\ast }}}$$

The *r*_outer_ is the radius of the entire particle (shell + core) and the *r*_inner_ is the radius of the core; ε*_outer_ and ε*_inner_ are the complex permittivities of the shell and core, respectively.

To characterize the anti-clogging method, we calculated the real part of the Clausius-Mossotti factor of blood. The relative permittivity and electric conductivity of blood plasma are 78 and 60 mS/m, and those of a blood cell are known to be 63 and 1.0 µS/m, respectively^28,32^. The real part of the Clausius-Mossotti factor for blood cells in plasma as a function of the frequency was calculated using equations (5) to (7) (shown in Fig. 2d). The real part of the Clausius-Mossotti factor was about −0.5 for an applied electrical frequency of <10 kHz for blood cells. Therefore, to generate nDEP force, the frequency was set to 1 kHz.

To prevent clogging of the microchannel by cell adhesion, the repulsive force generated by DEP must be larger than the adhesive force acting on blood cells, such as WBCs, RBCs, and PLTs. The adhesive and the DEP forces were calculated using the equations (2) and (3), respectively. The parameters in the equations were derived from the general properties of the cell membrane and plasma. Figure 2b shows the adhesive and DEP force expressed as particle diameter range. When an electric potential was applied, the adhesive force increased linearly, whereas the DEP force increased exponentially. Moreover, an increase in the electric potential also elevated the DEP force. At the diameter range of blood cells (WBC: 10–15 µm, RBC: 7–8 µm, PLT: 2–3 µm), the DEP force was greater than the adhesive force. According to the calculated results, although the DEP force is not effective on cells less than 1 µm in diameter, it is greater than the adhesive force on cells more than 1 µm in diameter. Thus, the clogging of microchannels by blood cells is expected to decrease as the electric potential increases.

In addition, the relationship between the adhesive and DEP force expressed as the distance from the cell to the surface is important for the application of the anti-clogging technique. As shown in Fig. 2c, the adhesive force is high in the short distance, *h* < 0.5 nm. However, at *h* > 0.5 nm, the adhesive force is smaller than the repulsive DEP force for anti-clogging. In addition, when the electric potential for generating the DEP force is further increased, the cross point between adhesive and DEP force, i.e., the minimum distance of the anti-clogging technique, shifted to a shorter distance, improving the efficacy of the anti-clogging technique. As a result, the DEP force can disturb migration of the blood cells toward the surface of the microchannel wall at *h* > 0.5 nm, which prevents cell adhesion.

### Blood plasma separation

In a microfluidic channel, blood cells tend to migrate in the axial direction of the microchannel. Therefore, the cell concentration along the centre of the microchannel increases, and the cell-free region, which is a marginal zone devoid of cells along the microchannel walls, is formed concurrently, as shown in Fig. 1a. Moreover, when the blood cells flow through a bifurcating region, they have a tendency to move to the channel with the higher flow rate than to the channel with the lower flow rate, as shown in Fig. 1a. The phenomenon is called the bifurcation law. The asymmetrical distribution of pressure and shear forces on the cell at the bifurcation pulls it towards the channel with the higher flow rate^32,33^. Thus, in a microfluidic channel, cells are drawn into the microchannel with a higher flow rate because they are subjected to a higher pressure and a torque which is produced by the asymmetric distribution of shear forces^33,34^. In conclusion, when the blood cells migrate through the microchannel, the cell-free region is formed near the channel wall and the blood plasma can be separated using the bifurcation law, without the cells moving to the branch channel. However, some cells migrate to the branch channel unwantedly; these attaches to the narrow branch channel and promote clogging.

In this study, plasma separation was accomplished by designing a simple microfluidic network based on the bifurcation law and DEP force for the anti-clogging. Because of the DEP force which disturbed cell migration to the branch channel for extracting the blood plasma, we can use relatively wide branch channels as compared with previous hydrodynamic blood plasma separation devices. That ensures a higher plasma yield. Moreover, separation of pure blood plasma (without PLTs and WBCs) from PLTs and WBCs contained blood, which was mostly not mentioned in reports about hydrodynamic blood plasma separation devices^5–8,10,11^, is also possible because of the DEP force.

## Results and Discussion

### Anti-clogging method and cell loss

**Figure 3:**
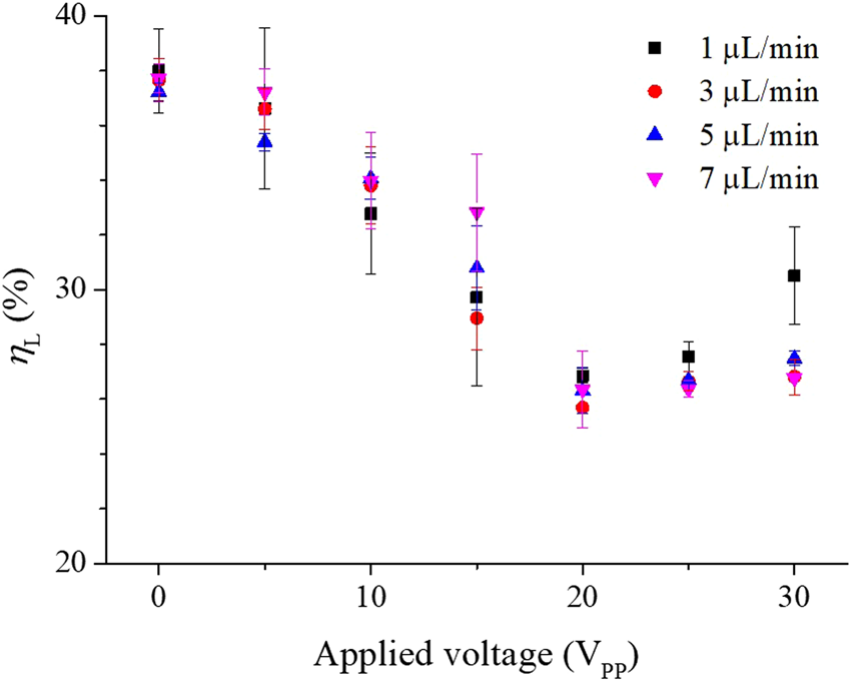
Optimisation of cell loss. The cell loss was plotted against applied voltage and flow rate at 15 min after the beginning of the experiment. An increase of the applied voltage caused a reduction in cell loss because the DEP force for the anti-clogging increased with the voltage. On the other hand, when the applied voltage was above 20 V, irreversible mechanical breakdown of the cell membrane happened, which led to the complete cell lysis. Therefore, the cell loss increased at voltages above 20 V.

To estimate the anti-clogging method, cell loss *η*_L_ was defined as

$${\eta }_{{\rm{L}}}=(1-\frac{{C}_{{\rm{o}}}+{C}_{{\rm{p}}}}{{C}_{{\rm{i}}}})\times 100\,( \% )$$

where *C*_i_, *C*_o_, and *C*_p_ is the original concentration of the blood cells at the inlet, the processed concentration at the blood outlet, and the concentration at the plasma product outlet, respectively. If the cell loss was high, adhesion of the blood cells to the microchannel wall was high. Therefore, when the cell loss was closed to zero, the anti-clogging method was working properly. Figure 3 shows the cell loss plotted against the applied voltage at different flow rates measured at 15 min after the beginning of the experiment. The cell loss was 37.98 ± 1.54 (mean ± S.D.) % and 37.71 ± 0.52% without an electric potential at the flow rate of 1 and 7 µl/min, respectively. When the electric potential was 20 V, the cell loss was 27.54 ± 0.25% at 1 µl/min and 26.35 ± 1.40% at 7 µl/min. In the 0 to 20 V range of the applied voltage, the change of the cell loss according to the flow rate was not significant. An increase of the applied voltage caused a reduction in cell loss because the DEP force for anti-clogging increased with the voltage. When the applied voltage was over 20 V, however, the cell loss was increasing again. At 30 V and 1 µl/min, the cell loss was 30.50 ± 1.77%. The increase of the cell loss at higher applied voltage was due to electroporation, which is a common method for creating transient pores in cell membranes. When the applied voltage is higher than the threshold voltage, irreversible mechanical breakdown of the cell membrane will happen, which leads to the complete lysis of cells. Therefore, above 20 V, some of the blood cells were lysed. When cell lysis increased, the concentration of the blood cells in the microchannel dropped. Accordingly, the detected cell loss increased at voltages above 20 V. Moreover, at a flowrate of 1 µl/min, cell lysis became severe because the exposure time for cells to the DEP force increased. We found that 20 V was the maximum voltage without cell lysis in the experimental device.

### Initial performance of the experimental device

**Figure 4:**
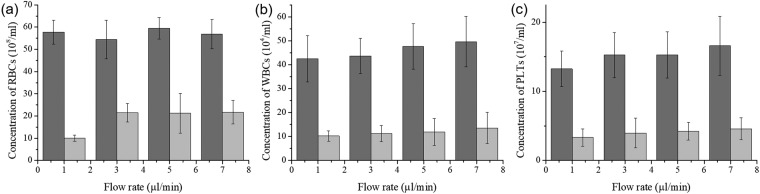
Blood cell removal with and without the anti-clogging method. The concentrations of (**a**) RBCs, (**b**) WBCs, and (**c**) PLTs in recovered plasma (collected at the plasma outlet) at 15 min after experiment initiation. Dark grey bars indicate data without the anti-clogging method; light grey bars indicate data with anti-clogging method performed at 20 V. The plasma in anti-clogging samples barely contained blood cells (RBCs, WBCs, and PLTs) compared with anti-clogging samples.

We monitored the concentration of blood cells in the processed plasma at 15 min after the beginning of the experiment. Figure 4a–c show the concentrations of RBCs, WBCs, and PLTs in separated plasma, respectively. When the applied voltage was 0 V (without anti-clogging method), the concentrations of blood cells were relatively high. However, the concentration of blood cells at 20 V (applied anti-clogging method) was low. By applying the anti-clogging method, the purity of the separated plasma increased. Moreover, the plasma, which was separated by our device at 20 V had low counts for WBCs and PLTs. Thus, using the experimental device, the difficult separation of poor WBCs and PLTs plasma from blood is possible.

**Figure 5:**
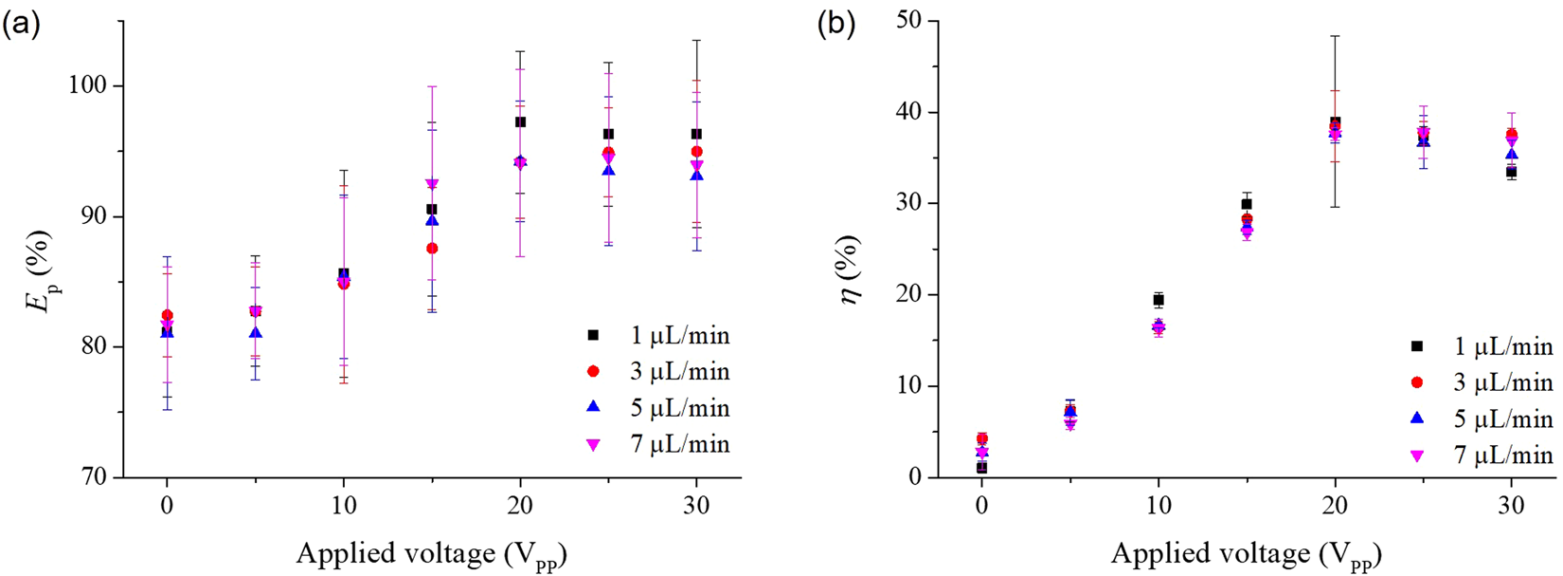
The purity efficiency and the plasma yield achieved with the experimental device. (**a**) The purity efficiency of the collected blood plasma at 15 min after the beginning of the experiments under applied voltage and flow rate in the range of 0 to 30 V and 1 to 7 µl/min, respectively. When the applied voltage was high (strong DEP force), the purity efficiency of collected blood plasma was high. However, at values higher than 20 V, the purity efficiency dropped because of cell lysis. (**b**) The plasma yield of the experimental device at 15 min after the beginning of the experiments under same condition as in (**a**). The plasma yield increased to high purity efficiency if a high voltage was used (20 V). When the applied voltage was higher than 20 V, cell lysis occurred, and the plasma yield dropped because of adhesion of cell fragment to the microchannel wall.

To evaluate the performance of the experimental device, purity efficiency and plasma yield, which are the conventional evaluation indexes, were used. The purity efficiency *E*_p_ and plasma yield *η* are defined as^35,36^:

$${E}_{p}=(1-\frac{{C}_{p}}{{C}_{i}})\times 100\,( \% )$$

$$\eta =\frac{(100-{H}_{{\rm{p}}})({H}_{{\rm{o}}}-{H}_{{\rm{i}}})}{(100-{H}_{{\rm{i}}})({H}_{{\rm{o}}}-{H}_{{\rm{p}}})}\times 100\,( \% )$$

where *H*_i_, *H*_o_ and *H*_p_ are the haematocrit levels at the inlet, at the blood outlet (concentrated samples), and at the plasma outlet (processed samples), respectively. To determine the haematocrit levels, centrifugation was used. Figure 5a presents the purity efficiency of the collected blood plasma at 15 min after the beginning of the experiment, under applied voltages and flow rates in the range of 0 to 30 V and 1 to 7 µl/min, respectively. The purity efficiency was 81.16 ± 5.00% and 81.72 ± 4.44% without an electric potential at flow rate 1 and 7 µl/min, respectively. When the electric potential was 20 V, the purity efficiency was 97.23 ± 5.43%, the maximum value of the proposed device, at 1 µl/min and 94.09 ± 7.19% at 7 µl/min. The purity efficiency improved as the applied voltage was increased within the 0 to 20 V range. The purity efficiency was saturated when the applied voltage was over 20 V. Similarly, the cell lysis increased and the concentration of blood cells dropped at a voltage higher than 20 V. Accordingly, the purity efficiency also dropped above the 20-V level. The saturated purity efficiency over 20 V has a tendency for higher values at low flow rates because the hydrodynamic force was low and the DEP force, which is preventing cell migration to the branch channels for plasma separation, was acting effectively. Therefore, the purity efficiency had a higher value at low flow rate.

Figure 5b is the plasma yield of the proposed device at 15 min after the beginning of the experiment as a function of the applied voltage and flow rate. When the flow rate was 1 µl/min, the plasma yield had a minimum value of 1.04 ± 1.21% without applied voltage. When the applied voltage was increased, the plasma yield increased to a maximum value of 38.95 ± 9.34% at 20 V and 1 µl/min. The plasma yield was also saturated at voltages over 20 V like the purity efficiency in Fig. 5a. When the applied voltage was higher than 20 V, cell lysis occurred and cell debris increased. The cell debris can easily attach and clog to the microchannel because of the small size (small particles were not blocked from clogging, see Section 2). Therefore, at high voltage (over 20 V), the plasma yield dropped because of the adhesion of the cell debris.

All the results of the cell loss, the purity efficiency, and the plasma yield did not change significantly in relation to the flow rate change. However, when the applied voltage was 20 V and the flow rate was 1 µl/min, the experimental device had the best results in the cell loss, the purity efficiency, and the plasma yield. Therefore, we set the values for applied voltage and flow rate were 20 V and 1 µl/min, respectively, in the time-dependent experiment for testing the long-term use.

### Time-dependent experiment

To expand the lifespan of the device, an anti-clogging method was applied. The range of the applied voltage without cell lysis was 0 to 20 V. Additionally, at 1 µl/min flow rate, the device had a maximum value of purity efficiency and plasma yield. Therefore, we set the applied voltage to 20 V and the flow rate to 1 µl/min for anti-clogging.

**Figure 6:**
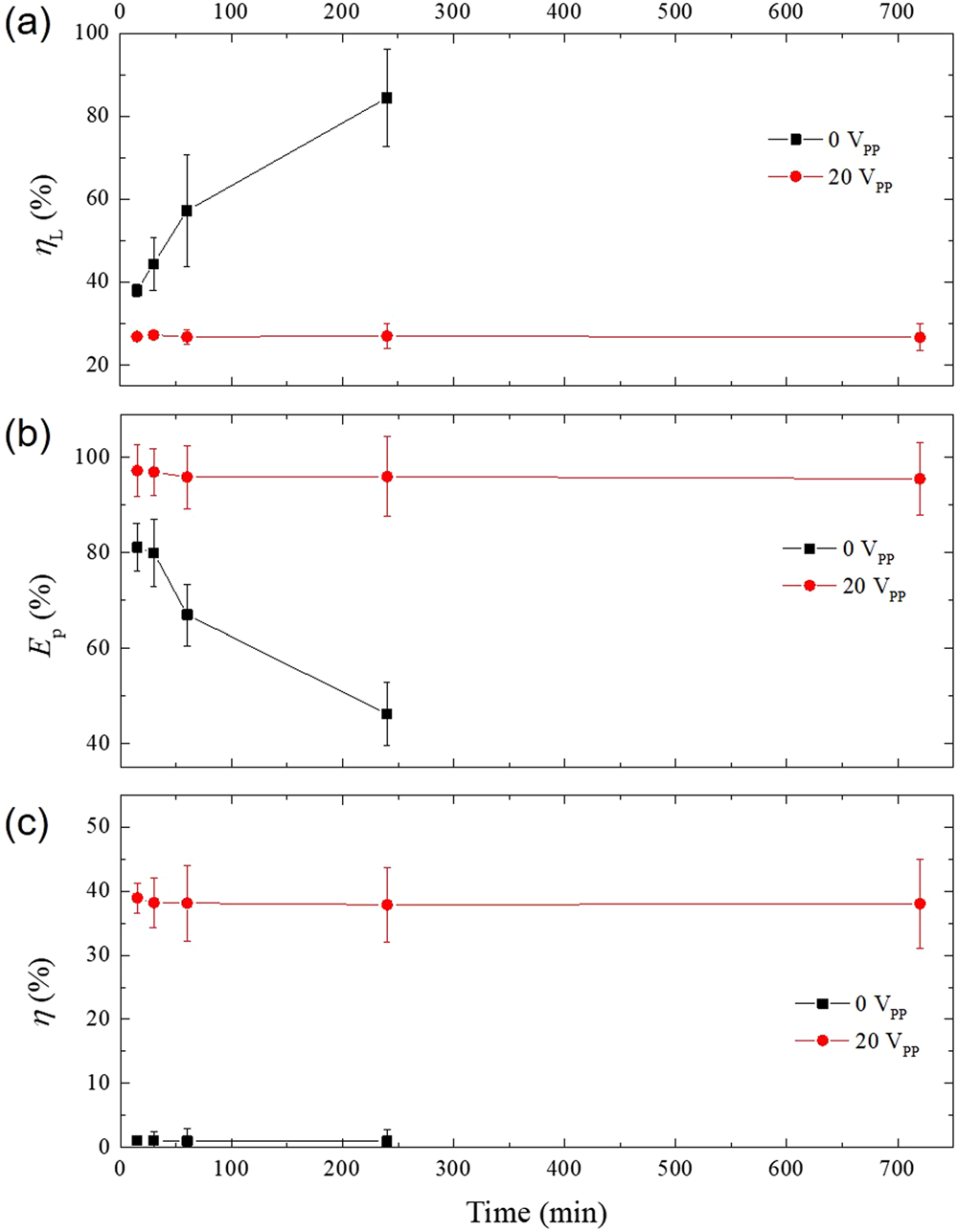
Performance parameters of the experimental device over time. (**a**) The cell loss, (**b**) the purity efficiency, and (**c**) the plasma yield over time (from 15 min to 12 h). The flow rate was 1 µl/min. Without the anti-clogging method (0 V), the cell loss increased over time. After 4 h, the attached cells fully clogged branch microchannels for the plasma extraction and the device did not work anymore. Similarly, the purity efficiency was dropped sharply and the plasma yield was very low until 4 h. In contrast, using the anti-clogging method (20 V), the cell loss, the purity efficiency, and the plasma yield were maintained over a much longer period (12 h).

Figure 6a presents the cell loss of the experimental device over time. We measure the cell loss at various time points, at 15 min, 30 min, 60 min, 4 h, and 12 h. When the applied voltage was 0 V, the cell loss was 37.99 ± 1.54% at 15 min. The cell loss was increased over time without the anti-clogging method. The cell loss without anti-clogging method was 84.47 ± 11.66% at 4 h. Then, over the 4 h, the cell loss was even higher, and the microchannel was fully clogged by blood cells. Therefore, the experimental blood plasma separation device was no longer working after 4 h. In contrast, when the anti-clogging method was applied (applied voltage of 20 V), the cell loss did not rise over time. The cell loss at 15 min and 12 h was 26.86 ± 0.226% and 26.68 ± 3.28%, respectively. Thus, cell clogging to the microfluidic channel wall was prevented by the anti-clogging method, and the device lifespan was increased due to the prevention of unwanted changes to the microchannel by clogging with blood cells. Figure 6b,c show the purity efficiency and the plasma yield of the experimental device in relation to the timeline of the experiment. Without applied voltage (without anti-clogging method), the purity efficiency dropped sharply from 81.16 ± 5.00% at 15 min to 46.25 ± 6.67% at 4 h. The plasma yield was 1.04 ± 1.21% at 15 min and 0.99 ± 1.74% at 4 h. Without the anti-clogging method, the plasma yield was very low. When the applied voltage was 20 V, however, the purity efficiency and the plasma yield did not decrease but maintained high values throughout the experiment. The purity efficiency was 97.23 ± 5.43% at 15 min and 95.52 ± 5.43% at 12 h. The plasma yield at 15 min and 12 h was 38.95 ± 2.34% and 38.03 ± 7.01%, respectively.

**Figure 7:**
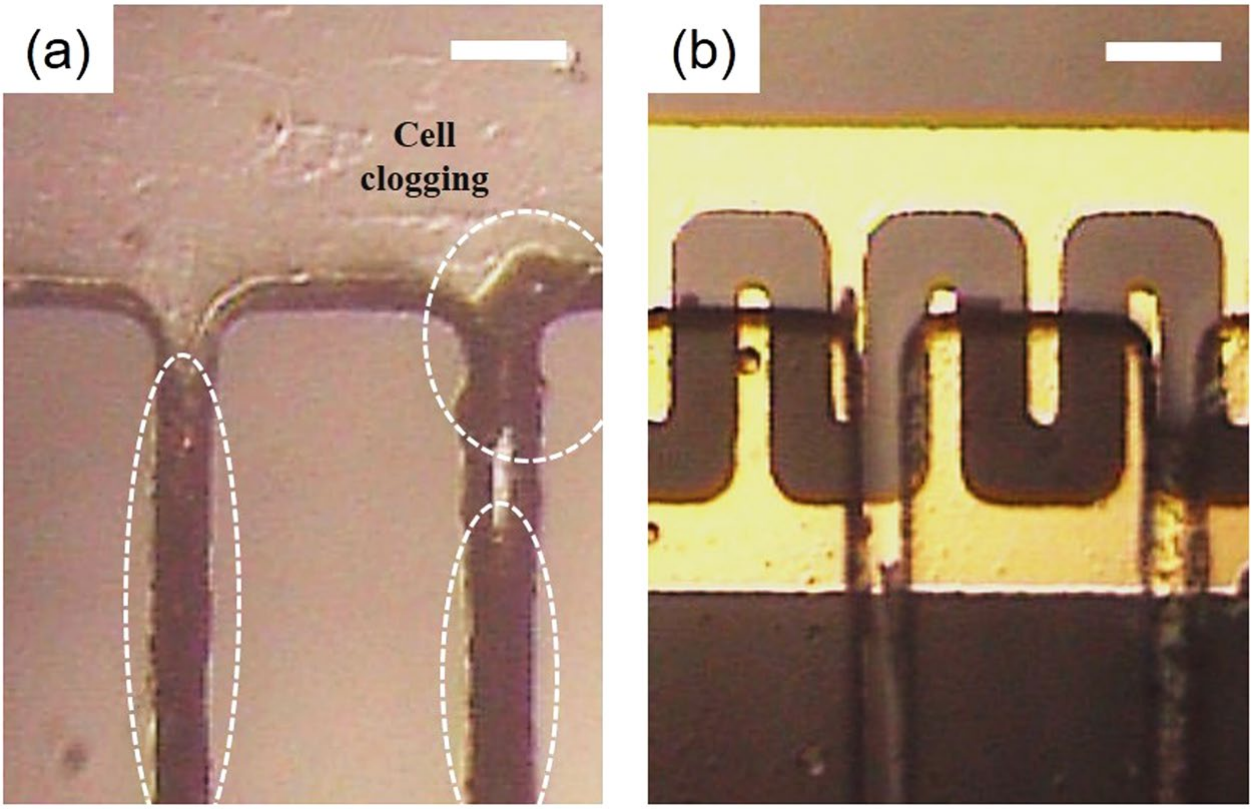
Assessment of microchannel clogging by image analysis. Images captured 4 h after the experiment initiation (**a**) without the anti-clogging method and (**b**) with the anti-clogging method (using DEP at 20 V). Without the anti-clogging method, the blood cells aggregated until the microchannel was fully clogged (t > 4 h). In contrast, when the anti-clogging method was applied, the blood cells were not attached and did not clog the microchannel wall. The scale bar is 50 µm.

The time-dependent experiment showed that using the applied voltage at 20 V was crucial for extending the lifespan of the experimental blood plasma separation device to at least 12 h without any cell clogging within the microchannel network. In addition, the performance of the experimental device, the purity efficiency, and the plasma yield were maintained over time. When the anti-clogging method was not applied, however, the microchannel was quickly clogged by the blood cells and the device stopped working at the 4-h time point (as shown in Fig. 7).

### Whole blood processing

**Figure 8:**
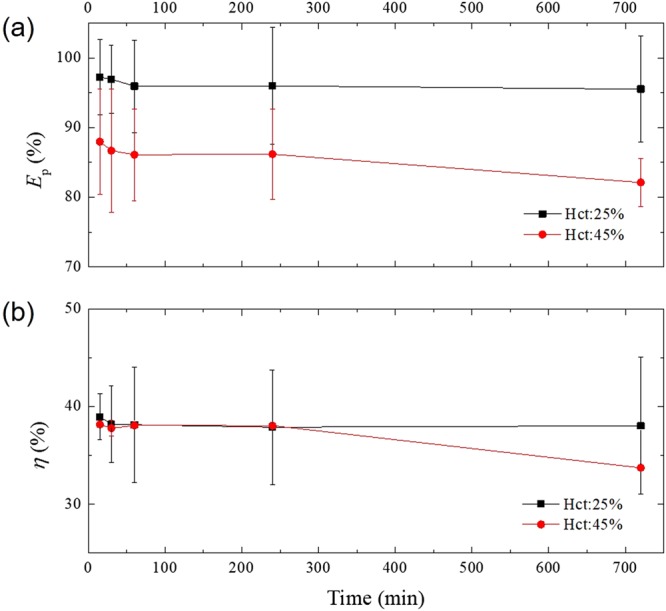
Application of the experimental device for whole blood processing. (**a**) The purity efficiency and (**b**) the plasma yield of the experimental device using diluted blood (Hct: 25%) and whole blood (Hct: 45%). The flow rate and the applied voltage were 1 µl/min and 20 V, respectively. After the whole blood sample was injected, the device continued working up to the last time point, 12 h. However, the purity efficiency was relatively low (82–88%) for whole blood processing.

To resolve the issue of diluting blood samples for on-chip device processing, we tested whether undiluted, whole blood can be processed using our experimental device. When whole blood was infused through inlet of the device at 1 µl/min, the purity efficiency was 87.96 ± 7.59% at 15 min and 82.12 ± 3.42% at 12 h (applied voltage, 20 V), as shown in Fig. 8a. The probability of cell adhesion was higher with whole blood than with diluted blood because of the high blood cell concentration in the undiluted sample. The purity efficiency of the whole blood sample started out low and gradually decreased over time. Figure 8b shows the plasma yield of whole blood test. The plasma yield at 15 min and 12 h was 38.18 ± 0.09% and 33.73 ± 0.01%, respectively. Thus, the plasma yield was low at the start and dropped over time. Interestingly, the experimental device was still working after 12 h. However, the performance of blood separation dropped over time. We confirmed the possibility of plasma separation using whole blood, but for clinical applications, a higher purity efficiency and plasma yield will be needed.

## Conclusions

Blood plasma separation is an important method because plasma is widely used to assess the health status of people. In step with the general progress in further developing the LOC technology, various on-chip plasma separators have been described. However, unwanted clogging by blood cell adhesion to the microchannel walls has been blocking any progress in this field. Clogging remains a major source of performance deterioration and, as a result, most plasma separators are disposable and not for long-term use or real-time monitoring, which is a limitation for expanding the on-chip technology. In this study, we applied an anti-clogging method for blood plasma separation to prevent unwanted cell adhesion to the microchannel wall and deterioration of device performance. When the anti-clogging method was applied, the purity efficiency, and plasma yield of the experimental device were 97.23 ± 5.43% and 38.95 ± 9.34% with diluted blood and 87.96 ± 7.59% and 38.18 ± 0.09% with whole blood, respectively, at 15 min after test initiation. In previous studies, when the high purity efficiency was achieved, the plasma yield was low and vice versa. For example, Tripathi *et al*.^37^ achieved a purity efficiency of 80%, but the plasma yield was only 3% with whole blood. Lee *et al*.^38^ achieved a plasma yield of 62.2%, but the purity efficiency was only 60% with whole blood. In contrast, the proposed device obtained both relatively high purity efficiency and plasma yield in real-time and continuous blood plasma separation with whole blood.

Over time and without the anti-clogging method, cell loss increased dramatically, eventually clogging the device, which did no longer work after 4 h. In contrast, when a voltage of 20 V was applied as an anti-clogging method, the experimental separation device was still working after 12 h without general performance deterioration, although a reduction in purity efficiency and plasma yield was observed. According to these results, we demonstrated that our anti-clogging method ensured the long-term use of an experimental on-chip device for plasma separation. We expect that the anti-clogging method can be applied for other blood plasma separators composed of a main channel and narrow branch channels. The experimental device with anti-clogging function is a promising candidate for usage in real-time and continuous blood plasma separation systems, because of its extended lifespan.

## Methods

The following sections provides a brief description of design of proposed device, fabrication techniques, and the experimental procedure. All the blood samples in this study were obtained with informed consent from all subjects. All the experiments in this study were performed in accordance with the guidelines and regulations which were approved by the bio-safety committee, Yonsei University, Korea.

### Design

The proposed blood plasma separation was accomplished using a microfluidic channel device specifically designed for blood processing. The details of proposed blood plasma separator are shown in Supplementary Fig. S2. The branch channels were positioned on each side of the main channel to separate the blood plasma. The number of branch channels were 50 (25 branches on each side). The width of the branch channels for blood plasma extraction was 15 µm. For adjusting the anti-clogging method to a hydrodynamic blood plasma separation device, the electrode for applying the DEP force was placed at the bottom of the microchannel.

### Fabrication

To test the anti-clogging method and blood plasma separation technique, the interdigitated (IDT) electrodes and the microchannel device were fabricated (Supplementary Fig. S3). The proposed microfluidic channel for separation of the blood plasma was made of polydimethylsiloxane (PDMS) using conventional soft lithography techniques. PDMS can be used as microchannel because it is suitable for optical detection (transparent), non-cytotoxic, and easily molded into patterned substrate. To form a PDMS replica of the microchannels, SU-8 (SU-8 2050, MicroChem Co.) was spin coated on a 4-inch silicon wafer at 1,800 rpm for 30 s to form a photoresist film (thickness: 80 µm). After soft baking (first: 65 °C for 3 min, second: 95 °C for 9 min), the SU-8 was exposed to UV light by a mask aligner (Karl Suss MJB3 Mask Aligner). The SU-8 was post-exposure baked (first: 65 °C for 2 min, second: 95 °C for 7 min) before developing. The SU-8 was soaked in fresh SU-8 developer solution (SU-8 Developer, MicroChem Co.) for more than 15 min with agitation and rinsed by IPA. A PDMS prepolymer and curing agent (10:1 mixture, Sylgard 184; Dow Corning, MI) was poured onto the patterned SU-8 and cured at 70 °C for 1 h. To form the IDT electrodes for anti-clogging methods, Ti (adhesion layer) and Au (working electrodes) were deposited on glass substrate at a thickness of 50 nm and 500 nm, respectively, using an e-beam evaporator, and patterned using a conventional photolithography method. For passivation and prevention of electrolysis, a SiO_2_ layer was deposited using PECVD at a thickness of 100 nm. To complete the proposed blood plasma separation device, both the PDMS replica and the IDT electrodes on a glass substrate were aligned (Karl Suss MJB3 Mask Aligner) and bonded after oxygen plasma treatment.

### Blood sample

Fresh human blood from healthy donors was collected into an EDTA-2K coated vacuum tube to prevent the blood coagulation. The blood samples were pretested for any communicable diseases. Samples were stored at 4 °C and used within a week after harvest. The collected blood was diluted in a phosphate buffered saline (PBS) solution. The haematocrit of the diluted blood was measured adjusted by centrifugation at 25%. The haematocrit of whole blood was 45%.

### Experimental design

For the anti-clogging method, we used sinusoidal voltages ranging from 0 to 20 V generated with a function generator (CFG 253, Tektronix, USA) to create the DEP forces acting on blood cells. We used a syringe pump (KDS-410, KD Scientific, USA) to control the flow rate of the solution through the microchannel device. Before the experiment, the microchannel was rinsed by PBS. The microchannel during the experiments was monitored with a microscope and images were captured by a CCD camera (SOMETECH SV35, Republic of Korea). To determine the performance of the proposed device, a haemocytometer (Neubauer improved C-chip, INCYTO) was used to count cells at the inlet and outlets. All the experimental setup is shown in Supplementary Fig. S4. For counting WBCs and PLTs, blood and plasma was diluted with 1% ammonium oxalate solution to achieve an isotonic balance that only lysed RBCs, but not WBCs and PLTs.

## Electronic supplementary material

Supplementary Information

## Data Availability

All data generated during this study are included in this published article and its Supplementary Information files.
