# Supplementary material for: An anti-clogging method for improving the performance and lifespan of blood plasma separation devices in real-time and continuous microfluidic systems
Source: Sci Rep. 2018 Nov 19;8:17015. doi: 10.1038/s41598-018-35235-4 (PMC6242854; doi:10.1038/s41598-018-35235-4)
Supplement: Supplementary file 1 — Supplementary Information [file 41598_2018_35235_MOESM1_ESM.pdf]

An anti-clogging method for improving the performance and lifespan of blood plasma separation devices in real-time and continuous microfluidic systems

Dong-Hyun Kang, Kyongtae Kim & Yong-Jun Kim*

School of Mechanical Engineering, Yonsei University, 50, Yonsei-ro, Seodaemun-gu, Seoul 03722, Republic of Korea

*Corresponding author (E-mail: yjk@yonsei.ac.kr)

Supplementary Information

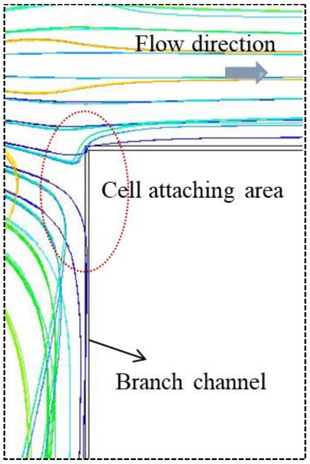

Fig. S1 Simulated results of the cell trajectories at the splitted microchannel.

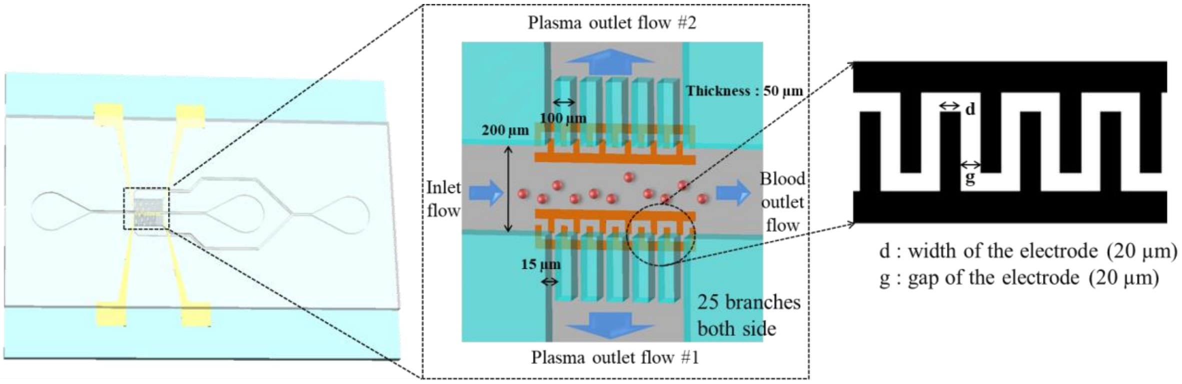

Fig. S2 Proposed microfluidic chips for blood plasma separation with anti-clogging method.

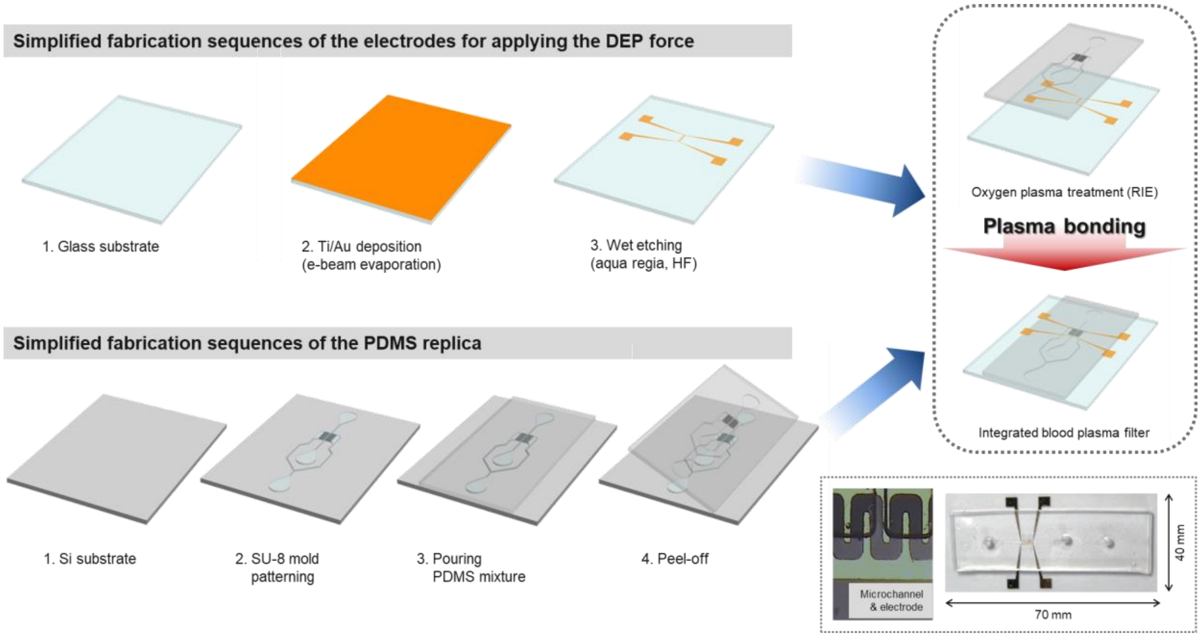

Fig. S3 Simplified fabrication flow charts of the proposed device

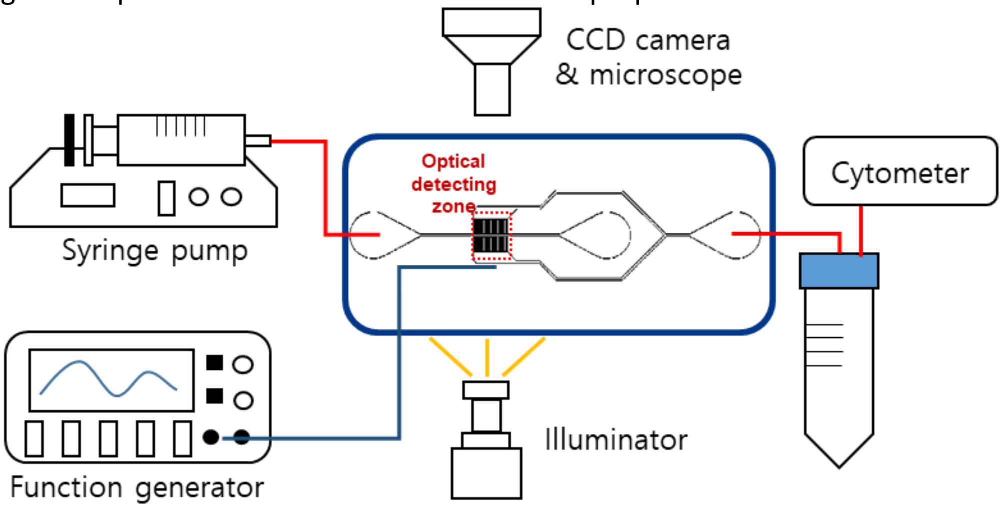

Fig. S4 Experimental setup for examining cell loss and plasma separation
